# Preparation and characterization of propolis reinforced eggshell membrane/ GelMA composite hydrogel for biomedical applications

**DOI:** 10.1186/s12896-023-00788-4

**Published:** 2023-07-11

**Authors:** Nahideh Asadi, Hadi Sadeghzadeh, Azizeh Rahmani Del Bakhshayesh, Amir Nezami Asl, Mehdi Dadashpour, Negar Karimi Hajishoreh, Sharif Kaamyabi, Abolfazl Akbarzadeh

**Affiliations:** 1 Department of Medical Nanotechnology, Faculty of Advanced Medical Sciences, Tabriz University of Medical Sciences, Tabriz, Iran; 2 Department of Tissue Engineering, Faculty of Advanced Medical Sciences, Tabriz University of Medical Sciences, Tabriz, Iran; 3 Health Research Center, Chamran Hospital, Tehran, Iran; 4 Department of Medical Biotechnology, Faculty of Medicine, Semnan University of Medical Sciences, Semnan, Iran; 5 Department of Chemistry, Farhangian University, Tehran, Iran

**Keywords:** GelMA hydrogel, Eggshell membrane, Propolis

## Abstract

Gelatin methacrylate-based hydrogels (GelMA) were widely used in tissue engineering and regenerative medicine. However, to manipulate their various chemical and physical properties and create high-efficiency hydrogels, different materials have been used in their structure. Eggshell membrane (ESM) and propolis are two nature-derived materials that could be used to improve the various characteristics of hydrogels, especially structural and biological properties. Hence, the main purpose of this study is the development of a new type of GelMA hydrogel containing ESM and propolis, for use in regenerative medicine. In this regard, in this study, after synthesizing GelMA, the fragmented ESM fibers were added to it and the GM/EMF hydrogel was made using a photoinitiator and visible light irradiation. Finally, GM/EMF/P hydrogels were prepared by incubating GM/EMF hydrogels in the propolis solution for 24 h. After various structural, chemical, and biological characterizations, it was found that the hydrogels obtained in this study offer improved morphological, hydrophilic, thermal, mechanical, and biological properties. The developed GM/EMF/P hydrogel presented more porosity with smaller and interconnected pores compared to the other hydrogels. GM/EMF hydrogels due to possessing EMF showed compressive strength up to 25.95 ± 1.69 KPa, which is more than the compressive strength provided by GM hydrogels (24.550 ± 4.3 KPa). Also, GM/EMF/P hydrogel offered the best compressive strength (44.65 ± 3.48) due to the presence of both EMF and propolis. GM scaffold with a contact angle of about 65.41 ± 2.199 *θ* showed more hydrophobicity compared to GM/EMF (28.67 ± 1.58 *θ*), and GM/EMF/P (26.24 ± 0.73 *θ*) hydrogels. Also, the higher swelling percentage of GM/EMF/P hydrogels (343.197 ± 42.79) indicated the high capacity of this hydrogel to retain more water than other scaffolds. Regarding the biocompatibility of the fabricated structures, MTT assay results showed that GM/EMF/P hydrogel significantly (p-value < 0.05) supported cell viability. Based on the results, it seems that GM/EMF/P hydrogel could be a promising biomaterial candidate for use in various fields of regenerative medicine.

## Introduction

Nano-biotechnology as a multidisciplinary science has provided innovative nano-carriers to overcome the mentioned restrictions in the realm of effective drug delivery, cell therapy, and regenerative medicine [1–4]. Nanocomposite materials with excellent features such as biocompatibility, simplicity of synthesis, and surface functionalization have gotten beyond the current state of the art in the context of nanomedicine and pharmaceutical science [5, 6]. Hydrogels have found wide applications in regenerative medicine. This is due to their inherent hydrophilicity and three-dimensional (3D) structure, which can create designs similar to natural extracellular matrix (ECM) in various aspects, especially water content and mechanical properties [4, 7]. Hydrogels derived from natural polymers such as collagen, gelatin, hyaluronic acid, etc., have wide biological applications due to their excellent cellular interactions. However, due to their structural degradation, poor mechanical properties, and immunogenicity, synthetic polymers such as poly (2-hydroxyethyl methacrylate) (PHEMA), poly (ethylene glycol) (PEG), etc. are also used to improve the structural and mechanical properties of them [8–14].

Among hybrid hydrogels made of biological and synthetic polymers, gelatin methacrylate (GelMA)-based hydrogels have attracted much attention [15]. The first reason to consider GelMA is the low immunogenicity of gelatin due to the presence of small number of aromatic groups [16]. The second reason is possessing various biologically active motifs, including glycine-aspartic acid-arginine (RGD) [17] in the structure of gelatin, which promotes the cellular and matrix metalloproteinase (MMP) activities [18]. In addition to the above advantages, various physical and biological properties of GelMA hydrogels can be manipulated by setting up a synthetic process or adding different biomaterials, which makes them widely used in most tissue engineering applications [15].

Among the constituents that can be used to improve the various properties of hydrogels, nature-derived materials [19, 20], especially eggshell membranes (ESM) and propolis [21] have been considered for tissue regeneration purposes. Eggshell membranes are usually discarded as biological waste, unaware that these specific materials have extraordinary properties enable them to be used in various biomedical fields [1, 22]. Structurally, the ESM is a fibrous structure that can easily mimic the natural ECM [23]. On the other hand, the chemical structure of these fibers is mainly composed of proteins (85–85%) which include collagen (type I, V, and X) and glycoproteins, which make them suitable candidates for use in regenerative medicine [24]. Mechanically, the performance of ESM is similar to other biomaterials, which is another desirable feature. The composition and morphology of ESM are often improved using several methods, including modification of pore size and fiber density, thiolation, conversion to soluble eggshell membrane protein (SEP), and mineralization [25].

Propolis is another nature-derived substance that has long been used as a healing agent. Propolis is one of the widely used products of beekeeping, as a resinous substance produced by worker honey bees (Apis mellifera L.). 60% of propolis consists mainly of plant resins, while wax and pollens also comprise 30%. In addition to these, other components of propolis are Zn, Fe, fatty acids, enzymes, sugars, and vitamins. Polyphenols and essential oils are also very important components of this natural substance [26, 27]. Flavonoids, phenolic acids, and terpenoids in propolis are considered biologically active components, so the therapeutic effects of propolis are mainly attributed to the number of volatile components and polyphenols. Propolis is known as a natural antimicrobial agent, so it has been extensively studied in various fields of regenerative medicine, especially wound healing [28]. Propolis also has antiviral, antibacterial, antifungal, anticancer, antioxidant, and anti-inflammatory properties that depend on the origin and the local flora [29]. However, propolis alone cannot produce a long-term healing effect at the lesion site. Therefore, in various studies, hydrogels have been used as carriers to control the release of propolis to increase its effectiveness [30]. Some propolis-containing membranes, such as cellulose membranes, Latex membranes, Collagen films, and sodium alginate/gelatin films, have all been formulated with propolis and used for wound care purposes. Also, some hydrogels such as PVA, chitosan, and alginate have been used as propolis carriers [31].

Considering the extraordinary properties that propolis and eggshell provide, it is believed that their use in GelMA hydrogel will improve the biological properties and cause better tissue imitation, along with strengthening the physicomechanical properties. It is also expected that the presence of nature-derived materials such as eggshells and especially propolis in GelMA hydrogel will improve the physicochemical properties and healing potential of it.

Hence, this study aims to develop a new composite hydrogel based on GelMA hydrogel and natural biomaterials (eggshell membrane (ESM) and propolis) used in regenerative medicine. In this regard, GelMA was first synthesized by the interaction of gelatin and methacrylic anhydride, and the proton nuclear magnetic resonance spectrum (^1^ H NMR) was used to analyze the prepared product. The ESM powder was then prepared and suspended in GelMA solution, and the GM-EM hydrogels were made using visible light and cross-linking. After that, propolis-reinforced GM-EM hydrogels were prepared by immersing GM-EM hydrogel in propolis solution. Finally, the prepared hydrogels were evaluated in terms of biocompatibility and physicochemical structure.

## Materials and methods

### Materials

Gelatin (from porcine skin), methacrylic anhydride, Eosin Y, triethanolamine (TEA), and N-vinylcaprolactam (VC) were purchased from Sigma-Aldrich. NaOH, Dimethyl sulfoxide (DMSO) were supplied from Merck. Dulbecco’s Modified Eagle Medium (DMEM), Fetal bovine serum (FBS), Penicillin-Streptomycin (Pen/Strep), and Trypsin-EDTA were obtained from Gibco.

### GelMA synthesis

GelMA was synthesized by the reaction of type A porcine skin gelatin with methacrylic anhydride according to the previous study [32]. Briefly, 10% (w/v) gelatin solution was prepared under stirring in PBS at 50 ^º^C. Then, methacrylic anhydride (4 ml) was added to the gelatin solution (drop by drop under constant stirring) and allowed to react at 50 ^º^C for 3 h. After diluting the obtained solution with PBS, the final solution was dialyzed against deionized water at 40 ^º^C using 12–14 kDa cutoff dialysis membranes for 5 days and followed by freeze-drying. The obtained synthesized GelMA was stored at -20 ºC until further use. H-NMR spectroscopy was used to assess the successful synthesis of GelMA.

### Preparation of fragmented ESM fibers

The fragmented ESM fibers were obtained similarly to the previously described procedure [33] with some modifications. Briefly, the ESM was carefully removed from the eggshell and washed with deionized water to eliminate any impurities. The obtained ESM was incubated in 8 M acetic acid for 12 h under shaking vigorously and followed by drying at room temperature. After that, ESM was cut into small pieces and placed in NaOH (1 M) at 50 ^º^C and under vigorous stirring for 3 h. Then, the resulted fragmented ESM fibers were separated from the NaOH solution and lyophilized via freeze drying. Finally, the dried fragmented ESM was grinded to obtain a powder form of it.

### Fabrication of GM/EMF hydrogel

GM/EMF hydrogel was prepared according to the previous study with some modifications [34]. To fabricate GM/EMF hydrogel, GelMA (10%) solution was prepared in PBS containing 0.75 wt% TEA (as co-initiator) and 1.25 wt% N-vinyl caprolactam (as monomer) at 50 ^º^C under gentle shaking. After obtaining the GelMA solution, Eosin Y (0.5 mM) as the photoinitiator was added to it in the ratio of 1:4, respectively. The fragmented ESM powder (25 mg/ml GelMA hydrogel precursor) was suspended in the GelMA solution and crosslinked using visible light for 300 s. Also, GelMA hydrogel was prepared without ESM according to the above instruction. The resulted prepared hydrogels were soaked in deionized water for 15 min to eliminate the unreacted working groups.

### Fabrication of propolis-reinforced GM/EMF hydrogel

To prepare GM/EMF/P scaffolds, GM/EMF hydrogels were fabricated according to the previous section. The fabricated GM/EMF hydrogel was immersed in propolis solution (5% v/v in water/ethanol mixture) and shaked for 24 h at 37 ^º^C to get GM/EMF/P composite hydrogel. Then, the GM/EMF/P hydrogel was removed from the propolis solution and soaked in deionized water (37 ^º^C) for 15 min to eliminate the excessive propolis. Finally, the obtained GM/EMF/P hydrogel was lyophilized for characterizations.

### Physicochemical characterization of hydrogels

#### H^1^-NMR spectroscopy of GelMA

To confirm the successful synthesis of GelMA, H^1^-NMR spectroscopy (Bruker, DRX-500) was carried out using D_2_O as the solvent. For this purpose, 40 mg of the freeze-dried GelMA was dissolved in D_2_O and H^1^-NMR spectra was recorded by 400 MHz Spectrometer.

#### FT-IR spectroscopy

The specific chemical groups of EMF, GM, GM/EMF, GM/EMF/P, and propolis were characterized using Fourier transform infrared spectroscopy (FT-IR, Bruker, TENSOR 27, Germany). For this, the freeze-dried powder samples were mixed with KBr and compacted into pellets. Finally, the FT-IR spectra were recorded in the range of 500–4000 cm^− 1^.

#### Thermogravimetric analysis (TGA)

Thermogravimetric analysis of the fabricated hydrogels can determine the different material effects on the thermal stability of the hydrogels. The thermal stability of the fabricated GM, GM/EMF, and GM/EMF/P hydrogels was assessed by TGA (LINSEIS SPA PT 1600 device, Germany). The samples were heated from 25 ^º^C to 700 ^º^C under nitrogen flow and a heating rate of 10 ^º^C/min. The thermal decomposition behavior of the various scaffolds was assessed.

#### Morphology characterization

Scanning electron microscopy can be used to determine the surface properties and morphological changes of the fabricated hydrogels. Hydrogel porosity and pore size were investigated by this method. For SEM characterization, ESM and freeze-dried EMF, GM, GM/EMF, and GM/EMF/P were coated with a thin layer of gold and imaged by scanning electron microscopy (FE-SEM, MIRA3, Tescan). The average pore size of the fabricated hydrogels was determined using Image J software.

#### Water contact angle measurement

The wettability of the fabricated hydrogels was assessed by the water contact angle measurement method (Dataphysics OCA15 plus, Germany). In this method, a droplet is placed on the surface of the hydrogel and then the angle between the sample and the droplet is measured. This angle is called the contact angle, which is indicated as *θ.* For this, a small amount of water (4 µl) was dropped on the surface of the samples and the average contact angles were obtained using a camera and then measured by the image analysis software.

#### Swelling study

Different materials can influence the swelling properties of the fabricated scaffolds. Hydrophilicity evaluation of the scaffolds is essential as this property can improve cell attachment. To evaluate the swelling behavior of the fabricated GM, GM/EMF, and GM/EMF/P, the lyophilized hydrogels were weighted and incubated in PBS at 37 ^º^C for 24 h. At the predetermined time, the samples were removed from PBS and after eliminating their excessive surface water with a filter paper, they were weighed. Finally, the swelling percentage of the hydrogels was calculated by the following equation:

W_2_ and W_1_ represent hydrogel’s final swelled and lyophilized weights, respectively.

$$Swelling\,(\% )\, = \,\frac{{W2 - W1}}{{W1}}\, \times \,100$$

#### In vitro degradation study

Evaluation of the degradation behavior of the scaffolds is one of the important considerations for successful tissue engineering. Various scaffolds formulations and fabrication methods can be effective in the degradation behavior of the hydrogels. The in vitro degradation behavior of the fabricated samples was assessed until the 15th day by incubating them in PBS at 37 °C. For this purpose, the lyophilized hydrogels were weighted (W_i_) and immersed in PBS (pH: 7.4). After 5, 10, and 15 days of incubation, the hydrogels were removed from PBS, freeze-dried, and weighted (W_f_). The weight loss (%) of the fabricated hydrogels was calculated by the following equation:

$$Degradation\,\,(\% )\, = \,\frac{{Wi - Wf}}{{Wi}}\, \times \,100$$

#### Mechanical characterization

The mechanical properties are of essential importance in the development of hydrogels for tissue engineering applications. The mechanical properties of the GM, GM/EMF, and GM/EMF/P hydrogels were studied through compressive stress–strain measurements by a universal mechanical testing machine (Instron® machine, Instron Z010, Zwick/Roell). For this, hydrogels with cylindrical shape (11 mm in diameter and 60 mm in height) were prepared and placed in the plates and compressed at a compression rate of 5 mm/min until the samples were completely compressed. Mechanical properties such as compressive strength and Young’s modulus were investigated.

#### Biocompatibility study

The standard MTT assay was used to evaluate the cytotoxicity of the fabricated hydrogels by using mesenchymal stem cells (from adipose tissue of rat). The cells were cultured in DMEM containing 10% (v/v) FBS and 1% (v/v) Pen/Strep and incubated at 37 °C under 5% CO_2_. After reaching the suitable confluence, the cells were used for MTT assay. The prepared hydrogels were cut into circular small pieces and UV-sterilized for 1 h. The sterilized hydrogels were placed into the 48-well plate and cells (with a density of 5000 cells/well) were introduced to the hydrogels and empty wells (as control). At the predetermined time points (2, 5, and 7 days after cell seeding), DMEM of the wells was removed and after the addition of MTT solution (3 mg/ml PBS), the cells were incubated for 4 h at 37 °C. After this time, the MTT solution of each well was removed carefully and replaced with DMSO. The produced formazan crystals were dissolved by adding DMSO to the samples. Finally, the optical density (OD) of the dissolved formazan crystals was measured by a spectrophotometer plate reader (Awareness Technologies Stat Fax 2100 Microplate Reader) at 570 nm.

### Statistical analysis

For the statistical analysis, GraphPad Prism Software was employed. All the data were reported as mean ± SD. The one-way analysis of variance (ANOVA) test was used to determine the statical differences between groups. Statistically significant test results were reported by P-value < 0.05.

## Result and discussion

In the present study, we developed 3 types of hydrogels for tissue engineering applications. The fabrication process of hydrogels is shown in the graphical abstract. Firstly, GelMA was synthesized by gelatin and methacrylic anhydride in a established method. After synthesizing GelMA, GM hydrogel was prepared by photo-cross linking of GelMA reactive groups. For the fabrication of GM/EMF hydrogel, ESM was introduced to the GelMA precursor and then cross-linked by visible light. GM/EMF/P hydrogel was then obtained by the immersion of GM/EMF into the propolis solution. The fabricated hydrogels were characterized in terms of FTIR spectroscopy, TGA, surface morphology and microstructure, surface wettability, swelling, degradation, mechanical properties, and biocompatibility.

### Physicochemical characterization of scaffolds

#### H^1^-NMR spectroscopy of GelMA

**Fig. 1:**
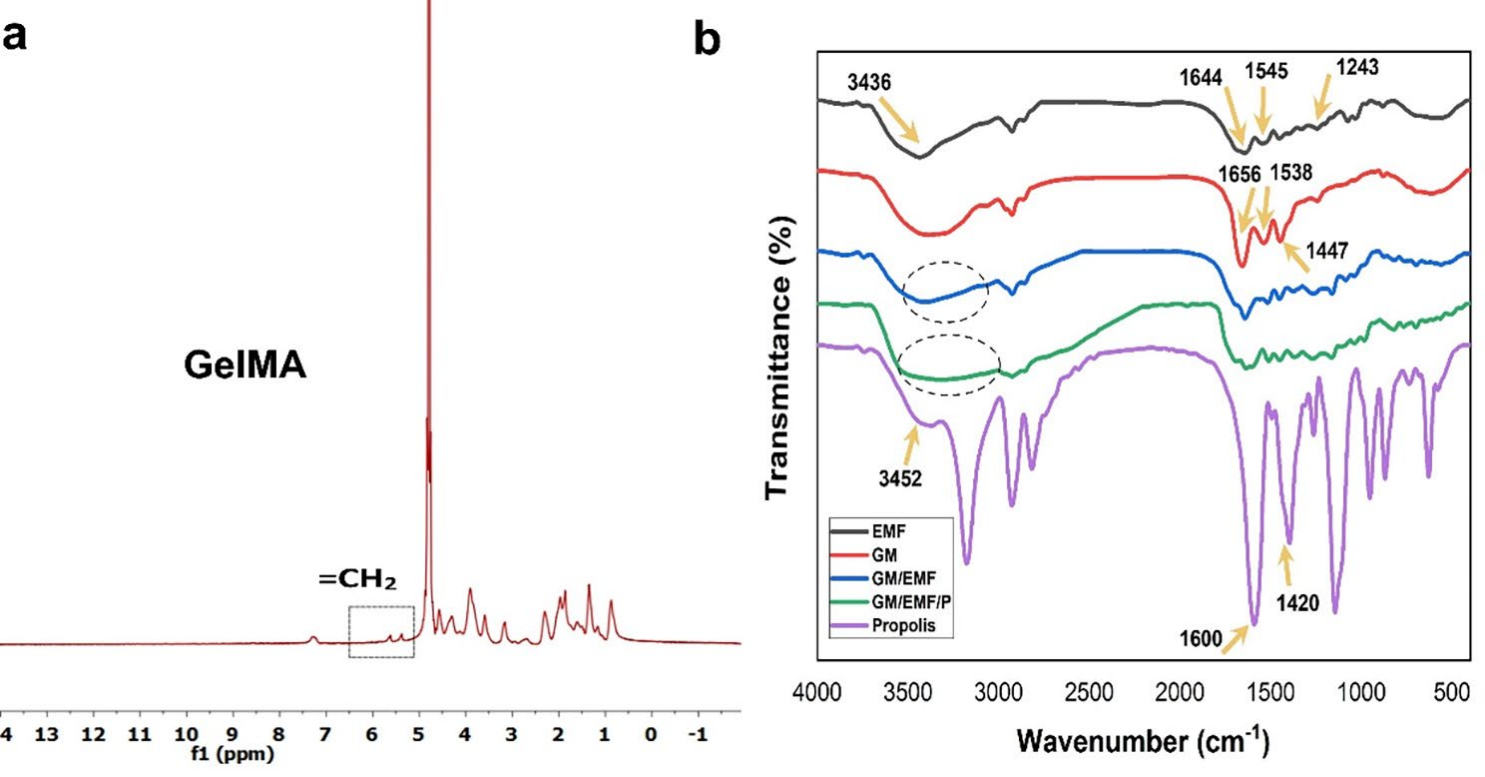
Characteristics of scaffolds. (**a**) ^1^ H-NMR spectrum of GelMA: the methacrylation of gelatin was showed by dashed line in the spectrum of GelMA. (**b**) FT-IR spectra of fragmented ESM fibers (EMF), GelMA hydrogel (GM), GM/EMF hydrogel, GM/EMF/P hydrogel, and propolis

The successful synthesis of GelMA via the grafting of methacryloyl groups to gelatin molecules was confirmed by H^1^_−_NMR spectroscopy. As shown in Fig. 1a, the presence of distinct peaks between 5 and 6 ppm was due to the acrylic protons of methacryloyl groups grafted to lysine and hydroxylysine residues of the gelatin backbone [35].

#### FTIR spectroscopy of fabricated hydrogels

FTIR spectroscopy was performed to study the chemical composition of samples. According to Fig. 1b, the FTIR spectrum of fragmented ESM showed the characteristic peak at 3436 cm^− 1^ for O–H and N–H stretching. Also, the transmittance peaks at 1644, 1545, and 1243 cm^− 1^ attributed to the amide I, amide II, and amide III, respectively [36]. About the GelMA hydrogel, the characteristic peaks at 1656, 1538, and 1447 cm^− 1^ were corresponded to amide I, amide II, and amide III, respectively. The presence of a broader peak near 3100–3500 cm^− 1^ in the spectrum of GM/EMF hydrogel corresponds to the O-H and N-H stretching. In the spectrum of propolis, the characteristic wide band near 3452 cm^− 1^ was attributed to the phenolic hydroxyl group (O-H) stretch of phenolic compounds. Also, propolis exhibited bonds at 1600 cm^− 1^ and 1420 cm^− 1^ that are related to the C = C stretches of aromatic rings [37]. The spectrum of GM/EMF/P, showed all the bonds of GM, fragmented ESM, and propolis.

#### TGA characterization

**Fig. 2:**
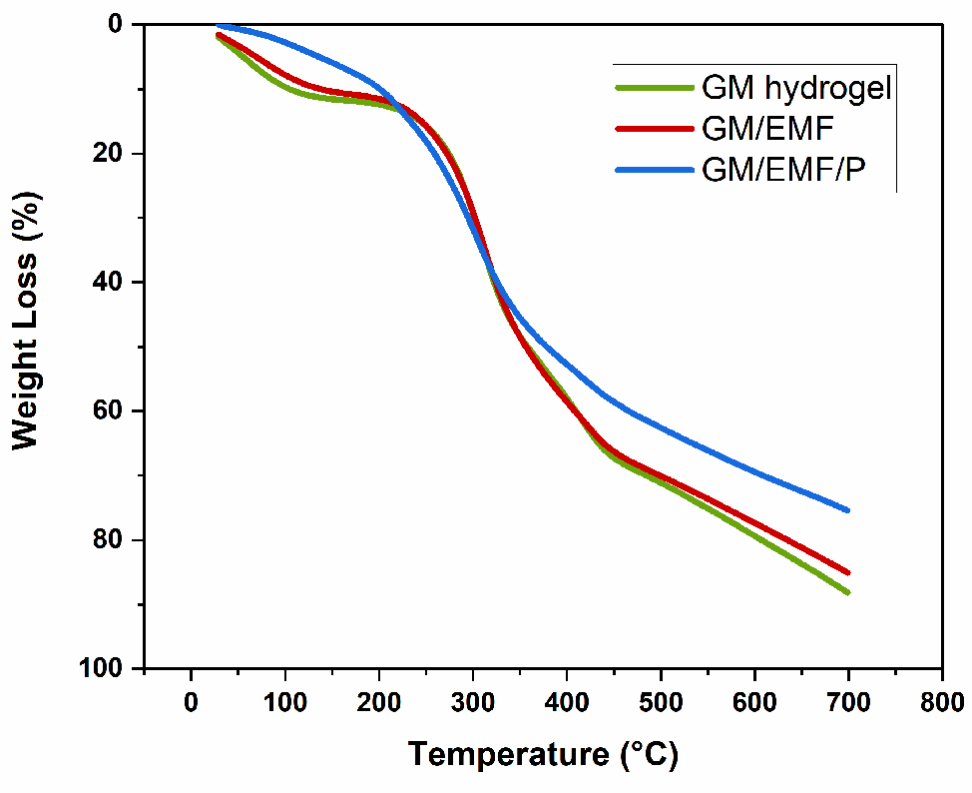
TGA plot of GM, GM/EMF, and GM/EMF/P hydrogels

The TGA analysis was carried out to assess the thermal stability of the prepared hydrogels. Figure 2 displays the TGA curves of GM, GM/EMF, and GM/EMF/P hydrogels. As shown in Fig. 2, the thermal gravimetric curves of all the samples exhibited three decomposition regions. Firstly, decomposition between 50 and 150 °C was associated to the physically adsorbed water by hydrogels. The second thermal decomposition was observed between 200 and 400 °C which can reveal the decomposition of GM, EMF, and phenolic compounds of propolis. The final decomposition region after 500 °C could be attributed to the inorganic impurities [38]. To calculate the thermal stability of the hydrogels, we explained the degradation temperature as a temperature at which 50% of the sample degraded was taken [39]. This temperature for GM, GM/EMF, and GM/EMF/P hydrogels was 358.224, 350.79, and 371.182, respectively. It can be shown that the incorporation of propolis into the GM hydrogel resulted in the thermal stability of the GM/EMF/P hydrogel. The residual weight in GM/EMF/P, GM/EMF, and GM hydrogels were 23.93%, 14.38%, and 11.84%, respectively. The interaction between fragmented ESM fibers and GM hydrogel can enhance the residual weight of GM/EMF hydrogel compared to GM [40]. The observed higher stability of the GM/EMF/P hydrogel could be originated from the formation of hydrogen bonding between OH groups of phenolic compounds of propolis and GM and fragmented ESM fibers functional groups that decrease the mobility of the polymer chains [41].

#### Morphological characterization

**Fig. 3:**
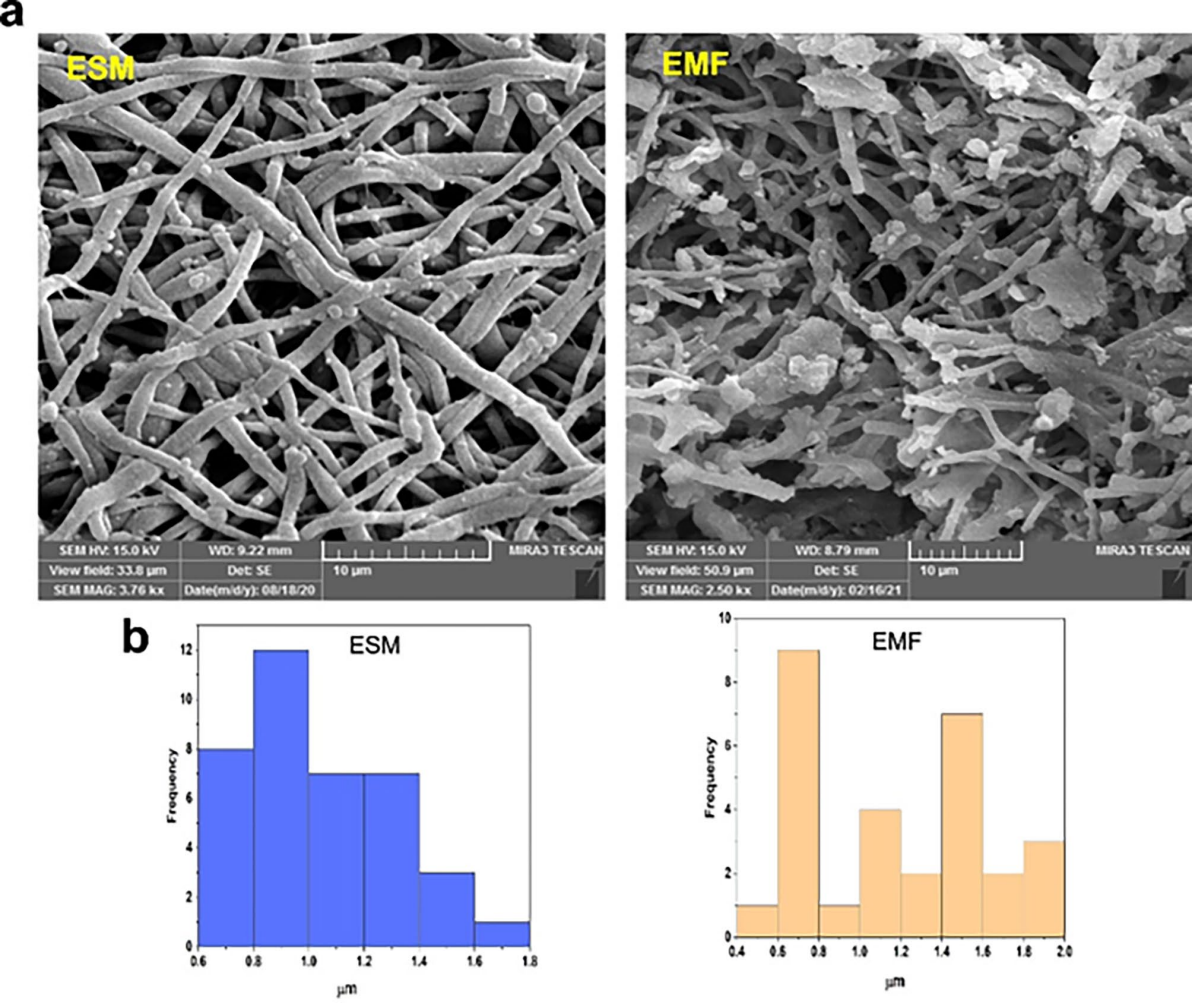
Characteristics of scaffolds. (**a**) FE-SEM images of ESM and EMF. (**b**) Diameter size distribution of ESM and EMF

**Fig. 4:**
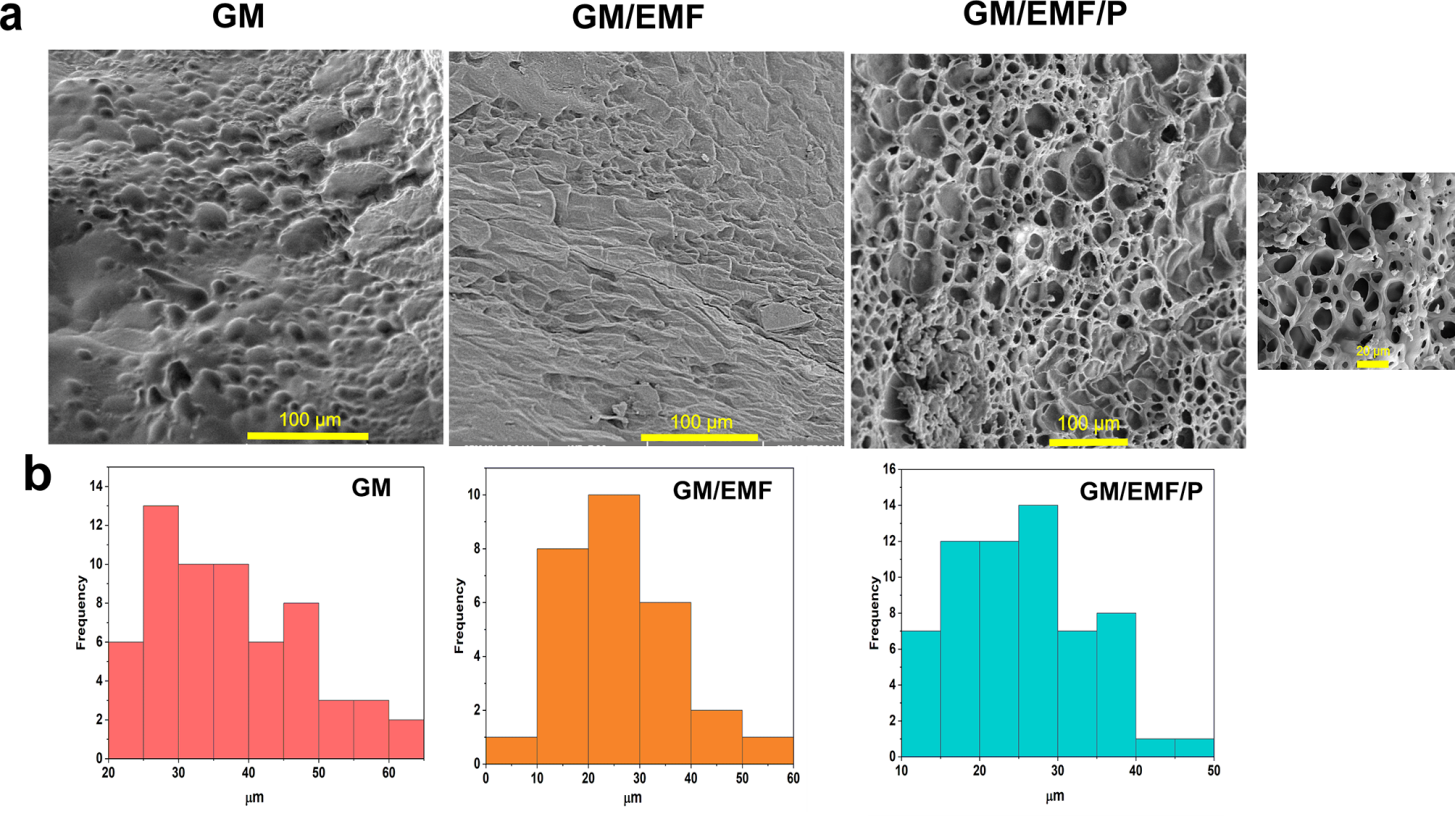
Characteristics of scaffolds. (**a**) FE-SEM images of GM, GM/EMF, and GM/EMF/P hydrogels. (**b**) Pore size distribution of GM, GM/EMF, and GM/EMF/P hydrogels

The morphology and microstructure of the fabricated hydrogels were investigated by SEM. The images of the fragmented ESM fibers, GM, GM/EMF, and GM/EMF/P hydrogels were illustrated in Figs. 3 and 4. The morphology and diameter size of the natural ESM and fragmented ESM fibers (after 3 h treatment with NaOH) are shown in Fig. 3. The ESM has lost own interwoven shape and broken to the shorter fiber fragments. It has been reported that the time of lysis reaction can alter the diameter and length of the fragmented fibers [42].

About the SEM image of the GM, GM/EMF, and GM/EMF/P hydrogels, differences in the morphology and pore size of them were shown in Fig. 4. All of the hydrogels exhibited a porous structure. The mean pore size of the GM hydrogel was 37.21 ± 5.95 when the GM/EMF that contain fiber showed a dense structure with smaller pores and protrusions on its own walls. Also, previous studies has been showed that the addition of nanofiber to the scaffold can lead to smaller pores of them [43]. As illustrated in Fig. 4a, the GM/EMF/P hydrogel presented more porosity with smaller and interconnected pores that could be a good candidate for cellular attachment and migration. The smaller pores of the GM/EMF/P hydrogel can be due to the coating of propolis on the network of hydrogel and hydrogen bonding with the functional groups of the GM and fragmented ESM fibers. The such a porous structure can be a good candidate for homeostasis at the wound site by providing enough gas and nutrient transport [44]. The porosity and pore size of the scaffold are considered as important factors that affect cell migration, vascularization potential, and cellular organization [45].

#### Water contact angle measurement

**Fig. 5:**
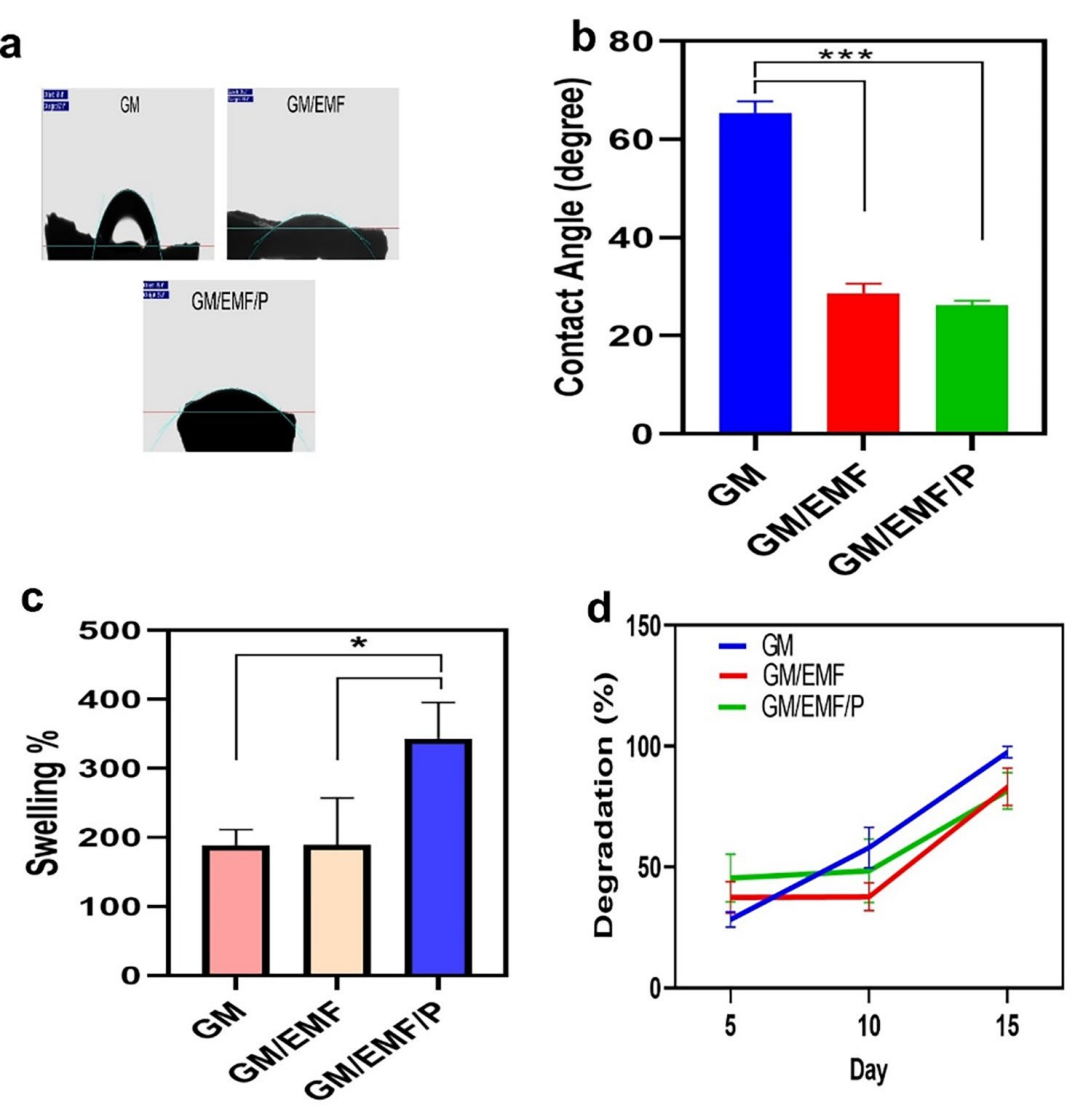
Characteristics of scaffolds. (**a**) and (**b**) Water contact angle images and graph for GM, GM/EMF, and GM/EMF/P hydrogels. (**c**) Swelling percentage of different scaffolds. (**d**) In vitro enzymatic degradation behavior of fabricated hydrogels. *P < 0.05, ***p < 0.001

The previous studies reported that the hydrophilicity of the scaffolds can influence the cell–scaffold interactions [46]. This interaction can influence cellular responses via cellular adhesion, migration, and proliferation. To evaluate the surface wettability of the prepared hydrogels, water contact angles were measured. The water contact angle images and mean size graph of the GM, GM/EMF, and GM/EMF/P hydrogels are illustrated in Fig. 5a and b, respectively. The contact angle results showed that GM hydrogel with a contact angle of about 65.41 ± 2.199 *θ* have more hydrophobicity compared to GM/EMF (28.67 ± 1.58), and GM/EMF/P (26.24 ± 0.73) hydrogels. The contact angle was decreased with the addition of EMF and propolis to the GM hydrogel. Mohammadzadeh et al. reported a decrease in the contact angle with the addition of a soluble eggshell membrane into the nanofiber [47]. The presence of a lot of functional groups (such as –COOH, –NH_2_) due to the possessing various collagen types I, V, and X in EM can be effective in the hydrophilicity of that. In the previous study, it was reported that enhancing the concentration of propolis exhibited a low contact angle and improved the hydrophilic nature of the scaffold [37, 48]. This improvement in the hydrophilicity of the scaffold can have a much better cellular attachment [37]. Also, in another study, the incorporation of propolis into the PVA and chitosan-based membranes could slightly decrease the contact angle [49]. It has been reported that propolis due to possessing phenolic components can improve the hydrophilicity of the scaffolds [50].

#### Swelling ratio

Hydrogel swelling behavior is another important parameter that should be considered in developing a scaffold for tissue engineering. Scaffolds with more hydrophilicity can provide a favorable environment for cell infiltration and migration. Also, a hydrated scaffold can facilitate the diffusion of small molecules such as nutrients and waste for the cellular process [51]. The swelling property evaluation can be an effective factor to predict the degradation behavior of the hydrogel. So, we determined the swelling percentage of GM, GM/EMF, and GM/EMF/P hydrogels after 24 h immersion in PBS. Normally, it was found that GM hydrogel due to its abundant hydrophilic groups has a higher water uptake capacity. The addition of EMF to the GM hydrogel had little effect on water uptake which may be due to the lower pore size of the GM/EMF. As the porosity and pore size of the hydrogels influences the water uptake capacity of the scaffolds. Hydrogels with small pores due to the formation of inner polymer networks can restrict the diffusion of water molecules to the scaffold [52]. The swelling property of the less porous structure of GM and GM/EMF hydrogels can be depend on the osmotic pressure [53]. Another reason for the lower swelling percentage of GM/EMF compared to GM/EMF/P may be due to the formation of hydrogen bonds between GM and EMF functional groups [54]. As shown in Fig. 5c, the GM/EMF/P presented a significantly higher swelling ratio compared to other groups, suggesting that propolis coating can improve the hydrophilicity of the hydrogel. This result confirms the contact angle measurement. The high swelling ratio of GM/EMF/P compared to other hydrogels can be originated from the more porous structure of this hydrogel.

#### Degradation

The degradation behavior of the scaffold is an important factor that can affect the successful healing of the tissues. Having an appropriate biodegradation rate for the replacement of newly formed ECM and tissue is an effective parameter that should be considered [55]. Degradation of scaffold supports cells to have enough space for the deposition of ECM [34]. To investigate the degradation behavior of the prepared scaffolds at the predetermined times, their mass before and after immersion in PBS was measured. Figure 5d shows the degradation behavior of the GM, GM/EMF, and GM/EMF/P hydrogels at the fifth, 10th, and 15th days after incubation. The swelling properties of the hydrogels can facilitate their degradation process via providing more freedom for amorphous chains to move easily [55]. So, all of the fabricated hydrogels demonstrated a considerable degradation rate after 15 days. Another factor that affect the degradation rate is the porosity of the scaffold as the porous structure can provide more fluid diffusion into the scaffold [56]. The GM/EMF/P and G/EMF hydrogels exhibited slower degradation rate compared to GM. The GM hydrogel showed faster degradation rate until 15 days. After 15 days, GM hydrogels degraded more than 90% of their initial weight but GM/EMF and GM/EMF/P hydrogels showed almost 83% degradation. But there was no significant difference between groups. In the previous study [34] biodegradation of eggshell-microparticle reinforced GelMA hydrogels were investigated. In their results, it was observed that the addition of eggshell microparticles led to less degradation of hydrogel. This result may be originated from the interactions between polymeric chains of hydrogel and eggshell microparticles.

#### Mechanical behavior of the scaffolds

**Fig. 6:**
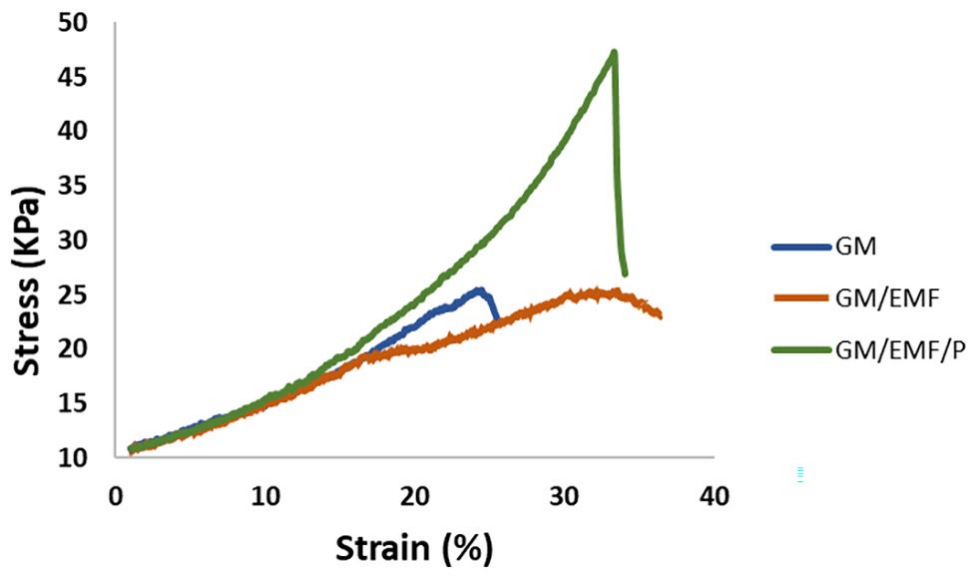
The stress-strain curves of the GM, GM/EMF, and GM/EMF/P hydrogels

Mechanical behaviors of hydrogel scaffolds are one of the important factors that influence the outcomes of tissue engineering. The mechanical properties of the hydrogels are presented in Table 1. Also, the stress-strain curves of the samples are illustrated in Fig. 6. The addition of the EMF into the GM hydrogel was investigated by measuring Young’s modulus and compressive strength. The compressive strength of the GM/EMF hydrogel was 25.95 ± 1.69 KPa which is more than GM hydrogel (24.550 ± 4.3 KPa). The presence of EMF in the GM/EMF hydrogels enhanced their compressive strength. This increase in the compressive strength of GM/EMF hydrogel was similar to the previous study [43]. The fiber tolerating against the mechanical stresses via absorbing, dispersing, and dissipating energy can be effective in the improved compressive strength of the GM/EMF hydrogel. Also, the smaller pore size of the scaffold can affect the mechanical strength [43]. The compressive strength of the GM/EMF/P hydrogel was considerably increased compared to the GM and GM/EMF. However, reverse results were observed between the Young’s modulus and compressive strength of GM, GM/EMF, and GM/EMF/P hydrogels. The Young’s modulus of GM, GM/EMF, and GM/EMF/P hydrogels were 2.29 ± 0.04 KPa, 1.95 ± 0.068 KPa, and 1.49 ± 0.2 KPa, respectively. This can most likely be attributed to the interaction between the polar components of propolis, especially phenolic acids and their esters, with the hydrophilic groups of GM hydrogel. These interactions that create stronger interfacial adhesion between the hydrogel and propolis molecules cause the tightening of polymer chain interactions and ultimately lead to more effective resistance of GM/EMF/P hydrogel to mechanical stress compared to GM and GM/EMF hydrogel. This is in accordance with what has been reported in previous studies [57]. According to various studies, the effects of such materials on the mechanical properties depend on the type and concentration of the additive, the macromolecule used, and the interaction between them [58]. However, there was a reverse result between the Young’s modulus of GM, GM/EMF, and GM/EMF/P hydrogels. It has been observed that with the addition of propolis and EMF, Young’s modulus, which indicates the degree of stiffness of the structure, has decreased. The higher Young’s modulus, the higher the stiffness of the construct. The decrease in Young’s modulus following the addition of propolis and EMF is probably related to the dispersion of components in the matrix, which caused the discontinuity of the structure and reduced the resistance of structures GM/EMF and GM/EMF/P to failure. This has also been stated in previous studies [58]. Another factor that caused a further decrease in Young’s modulus due to the presence of propolis in GM/EMF/P hydrogel is the waxes and essential oils that exist in the structure of propolis and act as softeners. As a result, the presence of propolis has increased the mobility of GM/EMF/P hydrogel chains and thus reduced their stiffness.

**Table 1:** The mechanical properties of the fabricated scaffolds

Samples	Compressive Strength (KPa)	Young’s modulus (KPa)
GM	24.550 ± 4.3	2.29 ± 0.04
GM/EMF	25.95 ± 1.69	1.95 ± 0.068
GM/EMF/P	44.65 ± 3.48	1.49 ± 0.2

#### Cell viability study

**Fig. 7:**
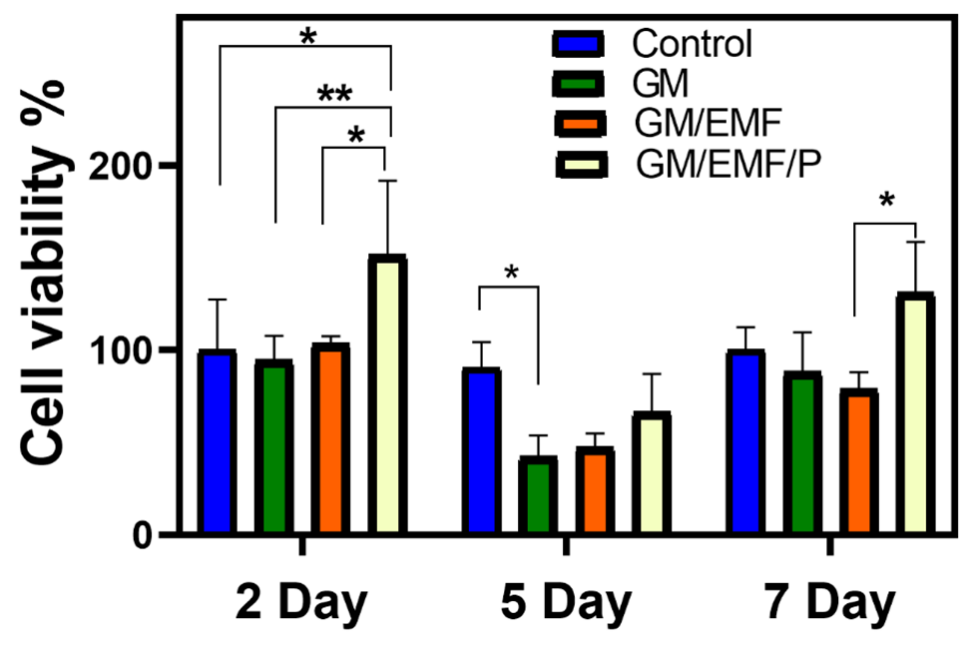
In vitro biocompatibility of mesenchymal stem cells on the scaffolds over 2, 5, and 7 days using MTT assay

The biocompatibility of the fabricated scaffolds is one of the critical parameters that should be considered in developing structures for tissue engineering. Also, the porosity of a scaffold can support cell viability via allowing the diffusion of the culture medium even without perfusion [59]. The application of a porous scaffold can provide advantages such as acting as ECM for cell attachment, proliferation, and differentiation [45]. Therefore, the cell viability and proliferation of the mesenchymal stem cells (from the adipose tissue of rat) was determined using MTT assay. Figure 7 shows the MTT results of the GM, GM/EMF, and GM/EMF/P hydrogels after 2, 5, and 7 days after cell seeding. As illustrated in Fig. 7, the incorporation of propolis in the hydrogel could significantly enhance the cell viability and proliferation on the second and 7th day of culture. This observed more biocompatibility in GM/EMF/P hydrogels can be due to the pro-proliferative nature of the propolis, as reported in the previous study [37]. Cell attachment as one of the important factors that affect biological responses is closely associated with the surface properties of the scaffolds [60]. The hydrophilic surface of the GM/EMF/P hydrogel may be effective in supporting cell proliferation. It can be concluded that the incorporation of propolis into the GM/EMF can improve cell viability.

## Conclusion

In summary, we have successfully fabricated a propolis reinforced composite hydrogel containing GelMA and fragmented ESM fibers for tissue engineering applications. The incorporation of EMF and propolis led to the thermal stability of the hydrogel. Also, the results indicated that GM/EMF/P hydrogel presents more porous structure and swelling ratio. In addition, the reinforcement of GM hydrogel with fragmented ESM fibers and propolis enabled it to have higher compressive strength compared to other groups. The cytocompatibility study by MTT assay exhibited more mesenchymal stem cells viability and proliferation in GM/EMF/P hydrogel. These data suggest that developed GM/EMF/P composite hydrogel due to providing appropriate physiochemical and biological properties can be an effective microenvironment for biomedical applications especially tissue engineering fields. It is suggested the differentiation of stem cells within the developed scaffold by focusing on the regeneration of damaged tissues such as bone, skin, and cartilage in future studies.

## Data Availability

The data that support the findings of this study are available from the corresponding author, upon reasonable request.

## Abbreviations

GelMA: Gelatin methacrylate-based hydrogels
ESM: Egg shell membrane
3D: three-dimensional
ECM: extracellular matrix
PHEMA: poly (2-hydroxyethyl methacrylate)
RGD: glycine-aspartic acid-arginine
MMP: matrix metalloproteinase
TEA: Triethanolamine
VC: N-vinylcaprolactam
DMSO: Dimethyl sulfoxide
DMED: Dulbecco’s Modified Eagle Medium
FBS: Fetal bovine serum
Pen/Strep: Penicillin-Streptomycin
